# Educational differences in the validity of self-reported physical activity

**DOI:** 10.1186/s12889-015-2656-7

**Published:** 2015-12-26

**Authors:** Annemarie N. E. Winckers, Joreintje D. Mackenbach, Sofie Compernolle, Mary Nicolaou, Hidde P. van der Ploeg, Ilse De Bourdeaudhuij, Johannes Brug, Jeroen Lakerveld

**Affiliations:** Department of Epidemiology and Biostatics, EMGO Institute for Health and Care Research, VU University Medical Center Amsterdam, De Boelelaan 1089a, 1081 HV Amsterdam, The Netherlands; Department of Movement and Sport Sciences, Ghent University, Ghent, Belgium; Department of Public Health, Academic Medical Centre, University of Amsterdam, Amsterdam, The Netherlands; Department of Public and Occupational Health, EMGO Institute for Health and Care Research, VU University Medical Center Amsterdam, Amsterdam, The Netherlands

**Keywords:** Accelerometer, Education level, Questionnaire, Self-report, Validity, Physical activity

## Abstract

**Background:**

The assessment of physical activity for surveillance or population based studies is usually done with self-report questionnaires. However, bias in self-reported physical activity may be greater in lower educated than in higher educated populations. The aim of the present study is to describe educational differences in the validity of self-reported physical activity.

**Methods:**

We included 196 healthy adults (age 57 ± 15.4, of whom 17 % low, 24 % medium and 59 % high educated). Criterion validity of an adapted International Physical Activity Questionnaire was assessed against the ActiGraph GT3X+ accelerometer.

**Results:**

While criterion validity of self-reported physical activity was low to moderate in the total sample (Spearman rho ranged from 0.16 to 0.27, depending on the variables used), the validity in lower educated respondents was poor (-0.07 to 0.05).

**Conclusions:**

The results confirm the hypothesis that self-report physical activity questionnaires are less valid in lower educated populations.

## Background

Lack of physical activity (PA) is an important health behaviour that has been posed as a risk factor for obesity, cardiovascular diseases and other chronic diseases [1]. Self-report questionnaires are most often used to assess PA at population levels. Although objective assessment methods such as accelerometry provide more accurate measures of PA, they are relatively expensive and hence not always practical in larger-scale cohorts [2]. Questionnaires are able to assess PA in different domains, while thus far accelerometers do not distinguish between transport-related physical activities, organised sports, household PA or occupational PA. Thus, a questionnaire may be preferred over (or next to) the use of accelerometers. However, reporting bias, including recall and social desirability bias, often leads to over-reporting and double counting of PA [3, 4]. Recalling PA is viewed as a highly complex cognitive task [5]. As typical PA questionnaires require respondents to recall and add-up different levels of PA in different domains over a period of time in the recent past, lower educated individuals may have more difficulties with interpreting and answering these questions. Moreover, there are indications that social desirability bias is larger in lower educated than in higher educated individuals. For example, a study among 81 US women showed that individuals who scored higher on the personality trait ‘social desirability’ had higher over-reporting of their PA levels, and lower educated women were more likely to have the ‘social desirability’ trait [6]. The validity of self-reported PA may therefore differ between educational groups.

However, it is not clear whether such educational differences exist. Systematic reviews on the validity of PA questionnaires do not mention differences between educational groups [7–10] while individual studies’ findings are equivocal. For example, in a population-based sample of 418 Swedish adults, low education was a significant predictor of overestimation of usual daily PA [11]. In a Dutch sample of 286 adolescents and 332 young adults, educational differences were observed in adolescents only, with lower educated individuals being less likely to over-report PA [12]. If over-reporting is consistent across educational groups, self-reported PA may still allow researchers to examine relative PA differences across educational groups. If not, the validity of self-report instruments may be compromised. Researchers may want to make an informed choice for using objective or subjective measures, based on the characteristics of their study population. In this study, we explored differences in self-reported and measured PA, and compared the validity of self-reported PA across educational groups.

## Methods

### Recruitment of participants

This study was conducted as part of the cross-European project ‘Sustainable prevention of obesity through integrated strategies – SPOTLIGHT’ [13]. A total of 6037 participants from 60 neighbourhoods in five countries participated in an online survey [14]. Of the 1609 Dutch participants who completed the survey, 379 left their phone number to be contacted between March and June 2014. Of those who could be reached (*n* = 305), 62 were not interested in participating and 41 did not meet the inclusion criteria (i.e. returning the questionnaire or informed consent, being 18 years or older and being able to walk one flight of stairs independently), resulting in a final sample of 202 respondents. The study was approved by the Medical Ethical Committee of the VU Medical Center and all participants to the survey provided informed consent.

### Measurements

#### Demographics

Respondents reported on their age, gender and education. The item on educational level was based on the Dutch Standard Classification of Education (SOI), which is comparable to the International Standard Classification of Education (ISCED) [15]. We divided the nine answering categories into low (no education/completed primary school/ lower vocational education/general secondary education), medium (secondary vocational or higher general secondary education), or high education (university bachelor-education degree or higher) as defined by ISCED.

#### Self-report physical activity

Participants were asked to complete a slightly adapted version of the International Physical Activity Questionnaire (IPAQ) long-form. The adaptations included combining two questions on moderate leisure time PA and vigorous leisure time PA into one. Combining moderate and vigorous PA was done to shorten the questionnaire, but also because the moderate and vigorous PA domains are commonly analyzed combined (MVPA). Data was cleaned for missing and out-of-range values according to the IPAQ scoring protocol ([16]) and two variables were derived: ‘time spent on MVPA’ (in minutes/day) and ‘time spent on total PA’ (in MET-minutes/day).

#### Objectively measured physical activity

Participants were asked to wear a tri-axial accelerometer (ActiGraph GT3X+), fixed to an elastic belt on the right hip, for seven consecutive days during waking hours. Non-wear time was defined as 60 min of consecutive zeroes, allowing for two interruptions of <100 counts per minute. Participants were included in the analysis if they wore the accelerometer for at least ten hours/day on at least five out of seven days. Accelerometer variables were averaged over valid days (wear time >10 h/day) and included ‘time spent in MVPA’ in min/day, ‘step counts/day’ and ‘total counts/day’.

### Statistical analysis

First, ANOVA was applied to compare patterns of self-reported (as measured with the IPAQ) and measured (with accelerometry) MVPA across educational groups. Second, to calculate criterion validity, IPAQ and accelerometry differences were tested using Pearson’s correlation coefficient – or Spearman’s correlation coefficient in case data was not distributed normally. Criterion validity was calculated for the total sample, as well as for the lower, medium and higher educated group. This allowed for the assessment of differences in criterion validity across educational groups (by eyeballing, as statistical differences between correlation coefficients are often not informative). We compared different self-report and accelerometry variables (the self-report measures ‘time spent in MVPA’ and ‘time spent on total PA’ with accelerometry measures ‘time spent in MVPA’, ‘step counts/day’ and ‘total counts/day’) to check for consistency of the findings.

## Results

Six of 202 participants were excluded from the study as they did not wear the accelerometer >10 h/day for at least five days. As a result, a total of 196 participants were included in this study (mean age 57 ± 15.4, 57 % women, of whom 17 % low, 24 % medium and 59 % high educated) (see also Table 1). Low, medium and high educated individuals did not differ significantly in either self-reported MVPA (188 min (SD ± 126), 193 min (SD ± 136) and 156 min (SD ± 118) respectively, *p* = 0.17) or objectively measured MVPA (26 (SD ± 23), 27 min (SD ± 24) and 31 min (SD ± 20) per day respectively, *p* = 0.32). All educational groups over-reported their MVPA, but this did not significantly differ between low, medium and high educated individuals.

**Table 1:** Characteristics of the study sample

	Total (*n* = 196)		Lower education (*n* = 33, 17.1 %)		Medium education (*n* = 47, 24.4 %)		Higher education (*n* = 113, 58.5 %)	
General descriptives	n	Mean (± SD) or number (%)	n	Mean (± SD) or number (%)	n	Mean (± SD) or number (%)	n	Mean (± SD) or number (%)
Age (years)	196	57.1 (±15.4)	33	61.0 (±14.5)	47	59.9 (±15.0)	113	54.78 (±15.5)
Gender	195		33		46		113	
-Men		84 (43.1 %)		18 (54.5 %)		17 (37.0 %)		48 (42.5 %)
-Women		111 (56.9 %)		15 (45.5 %)		29 (63.0 %)		65 (57.5 %)
BMI (kg/m^2^)	189	24.8 (±4.2)	32	26.0 (±4.9)	45	25.7 (±4.6)	110	24.2 (±3.7)
Ethnicity	192		33		47		110	
-Dutch		174 (90.6 %)		31 (93.9 %)		43 (91.5 %)		98 (89.1 %)
-Western immigrant		10 (5.2 %)		1 (3.0 %)		2 (4.3 %)		7 (6.4 %)
-Non-western immigrant		8 (4.2 %)		1 (3.0 %)		2 (4.3 %)		5 (4.5 %)

*SD* Standard deviation, *BMI* Body Mass Index

### Criterion validity

Table 2 shows the Spearman’s correlation coefficients for the comparison of the IPAQ and accelerometer. For the total sample, PA as measured with the IPAQ showed a relatively low correlation with PA assessed with the accelerometer (Spearman rho ranged from 0.16 to 0.27, depending on variables used). Stratified analyses showed that correlation coefficients were approximately five times lower in the low educated group compared to the medium and high educated group. The Spearman rho for lower educated individuals suggests that there was almost no agreement between rank in self-reported PA and objectively measured PA.

**Table 2:** Spearman correlation coefficients for the comparison of total physical activity as assessed with IPAQ and ActiGraph

Variables		Spearman correlation coefficients			
IPAQ (min.day^−1^)	ActiGraph (min.day^−1^)	All (*N* = 196)^d^	Lower education (*N* = 33)	Medium education (*N* = 47)	Higher education (*N* = 113)
MVPA	MVPA	0.157^*^	−0.029	0.328^*^	0.191^*^
MVPA	Step counts^a^	0.251^**^	0.054	0.350^*^	0.297^**^
Total PA^b^	MVPA	0.155^*^	−0.074	0.299^*^	0.200^*^
MVPA	Total counts^c^	0.268^**^	0.016	0.383^**^	0.322^**^

^a^ActiGraph total step counts

^b^IPAQ MET min.day^−1^

^c^ActiGraph counts.day^−1^

^d^For three people, information on educational attainment was missing. The sum of people in low, medium and high education therefore adds up to 193

*IPAQ* International Physical Activity Questionnaire, *MVPA* Moderate and Vigorous Physical Activity, *PA* Physical Activity

^*^ *p* < 0.05 ** *p* < 0.01

## Discussion

The results from this study confirm the pre-set hypothesis that criterion validity of self-reported PA is higher in the medium and high education group compared to the lower educated group. Whereas the validity in the high education group could be viewed as acceptable (low to moderate), the even lower validity in the low education group suggests that self-report results in a differential categorisation of individuals on the basis of education level.

We found overestimations of PA in all educational groups, concordant with a previous literature overview [4]. The IPAQ is known to result in over-reporting of PA [9], possible due to its’ asking for average times and best estimates of frequencies [17]. It has also been suggested that reporting issues are likely due to social desirability [6] or the inability to correctly assess the intensity level of an activity or to accurately recall time spent being active [18]. We did not find statistical significant educational patterns in over-reporting in our study, in contrast to other studies showing more over-reporting of PA in lower than in medium and higher educated individuals [11, 12]. The lack of significant educational differences in overestimation may have been due to the relatively small group sizes. This is in concordance with two other studies with small samples of low educated individuals: the study of Ekelund and colleagues did not find that education affected the correlation between self-reported and accelerometry-measured PA [12, 19], and the study of Rzewnicki concluded that over-reporters of PA were not more likely to be lower or higher educated [12, 17].

The study of Ekelund and colleagues did find that especially individuals with low levels of PA tended to overestimate their PA in a PA questionnaire [19]. It could be that overestimation of self-report PA is high in low educated groups with low levels of PA. The combination of low education and low levels of PA in this sample may therefore partly explain the lower validity for low educated participants as compared to those with higher educational attainment.

Despite lack of strong educational patterns in over-reporting of PA in this study, the validity of the self-report did seem to be affected by educational differences. This suggests that while medium and high educated individuals over-reported their PA, the self-report questionnaire was still able to rank individuals with low and high PA as such. For low educated individuals, the ranking of individuals according to their self-reported PA level did not correspond with the ranking according to measured PA. The reasons underlying the differential validity of PA self-report across educational groups should be further explored.

A strength of this study is that we included respondents across different educational strata. Our relatively small sample size could be viewed as a limitation, with 83 % of the participants corresponding to the medium or high education group. The relatively small sample of lower educated individuals may have limited the power to detect more pronounced educational differences in PA-specific self-report bias. Generalisation of the results to other PA questionnaires or populations should be done with caution as this study was performed in a Dutch adult population and we only evaluated (an adapted form of) the IPAQ long-form. Other questionnaires may show less education-specific bias.

## Conclusion

In conclusion, we found considerable educational differences in the validity of self-reported PA. These findings suggest that using a self-report questionnaire in the general population (i.e. with a range of educational backgrounds) might introduce a bias that will be stronger in lower educated respondents. Using objective measures to assess PA across a range of educational levels will generate better insight into overall PA levels, as well as improved identification of socioeconomic inequalities in PA.
